# Machine learning-based prediction of emergency neurosurgery within 24 h after moderate to severe traumatic brain injury

**DOI:** 10.1186/s13017-022-00449-5

**Published:** 2022-08-03

**Authors:** Jean-Denis Moyer, Patrick Lee, Charles Bernard, Lois Henry, Elodie Lang, Fabrice Cook, Fanny Planquart, Mathieu Boutonnet, Anatole Harrois, Tobias Gauss, The Traumabase Group®, Paer-Selim Abback, Gérard Audibert, Thomas Geeraerts, Olivier Langeron, Marc Leone, Julien Pottecher, Laurent Stecken, Jean-Luc Hanouz

**Affiliations:** 1 Department of Anesthesiology and Critical Care, Beaujon Hospital, DMU Parabol, AP-HP. Nord, 100 Boulevard du Général Leclerc, 92110 Clichy, France; 2 Capgemini Invent, Insight Driven Enterprise, Focused on Data and Artificial Intelligence Services, Paris, France; 3 Department of Anesthesiology and Critical Care, Lille, France; 4 Department of Anesthesiology and Critical Care, Hôpital Européen Georges Pompidou, Paris, France; 5 Department of Anesthesiology and Critical Care, Hôpital Henri Mondor, Créteil, France; 6 Department of Anesthesiology and Critical Care, Strasbourg, France; 7 Intensive Care Unit, Percy Military Teaching Hospital, 101 Avenue Henri Barbusse, 92140 Clamart, France; 8 Val de Grace Academy, Place Alphonse Laveran, 75005 Paris, France; 9 Department of Anesthesiology and Critical Care, APH-HP, Bicêtre Hôpitaux Universitaires Paris-Sud, Université Paris Saclay, Le Kremlin Bicêtre, France; 10 Déchocage- Bloc des urgences, Pole Anesthésie- Réanimation, CHU Grenoble Alpes, La Tronche, France

**Keywords:** Traumatic brain injury, Trauma, Emergency neurosurgery, Prediction models, Artificial intelligence

## Abstract

**Background:**

Rapid referral of traumatic brain injury (TBI) patients requiring emergency neurosurgery to a specialized trauma center can significantly reduce morbidity and mortality. Currently, no model has been reported to predict the need for acute neurosurgery in severe to moderate TBI patients. This study aims to evaluate the performance of Machine Learning-based models to establish to predict the need for neurosurgery procedure within 24 h after moderate to severe TBI.

**Methods:**

Retrospective multicenter cohort study using data from a national trauma registry (Traumabase®) from November 2011 to December 2020. Inclusion criteria correspond to patients over 18 years old with moderate or severe TBI (Glasgow coma score ≤ 12) during prehospital assessment. Patients who died within the first 24 h after hospital admission and secondary transfers were excluded. The population was divided into a train set (80% of patients) and a test set (20% of patients). Several approaches were used to define the best prognostic model (linear nearest neighbor or ensemble model). The Shapley Value was used to identify the most relevant pre-hospital variables for prediction.

**Results:**

2159 patients were included in the study. 914 patients (42%) required neurosurgical intervention within 24 h. The population was predominantly male (77%), young (median age 35 years [IQR 24–52]) with severe head injury (median GCS 6 [3–9]). Based on the evaluation of the predictive model on the test set, the logistic regression model had an AUC of 0.76. The best predictive model was obtained with the CatBoost technique (AUC 0.81). According to the Shapley values method, the most predictive variables in the CatBoost were a low initial Glasgow coma score, the regression of pupillary abnormality after osmotherapy, a high blood pressure and a low heart rate.

**Conclusion:**

Machine learning-based models could predict the need for emergency neurosurgery within 24 h after moderate and severe head injury. Potential clinical benefits of such models as a decision-making tool deserve further assessment. The performance in real-life setting and the impact on clinical decision-making of the model requires workflow integration and prospective assessment.

**Supplementary Information:**

The online version contains supplementary material available at 10.1186/s13017-022-00449-5.

## Background

Traumatic brain injury (TBI) is a major public health concern affecting mostly young people, victims of road traffic accidents, and the elderly, victims of falls [Using the random forest as the base model to train to get the importance of different features for selection]. Despite recent progress in neurosurgical and neurocritical care, TBI remains one of the most common causes of injury-related morbi-mortality [2]. In order to improve outcome, the Brain Trauma Foundation (BTF) Prehospital Guidelines recommend that TBI patients be transported to a hospital with computed tomography (CT) scanning, neurosurgical care and intracranial pressure (ICP) monitoring capacities [3]. Among moderate to severe TBI, 15–40% of patients will require an emergency intracranial surgery which including craniotomy and decompressive craniectomy [4–6]. These patients carry the higher risk of dying within the first 24–48 h due to extensive intracranial hemorrhage or severe intracranial hypertension and therefore require prompt identification and rapid referral to a specialized neurotrauma center (SNC) [7, 8]. Furthermore, the early identification of patients at risk of emergency neurosurgery (EN) will allow the trauma team to efficiently anticipate and prepare the required resources. This overall improvement could streamline the trauma system and improve time efficiency. However, identifying TBI patients who will benefit from EN remains challenging in the prehospital field, due to limited diagnostic resources in a miscellaneous pre-hospital environment [9]. Machine Learning (ML) provides a way to develop new clinical tools by exploiting large datasets and advanced computation resources [10, 11]. In recent years, many new clinical diagnostic tools have been developed using machine learning methods[12, 13]. This study aims to explore the use of machine learning to elaborate a predictive model of emergency neurosurgery within 24 h after hospital admission in patients with moderate to severe traumatic brain injury.

## Methods

### Study design

This is a retrospective, multicenter, cross-sectional diagnostic study that is conducted to predict the need for neurosurgery within 24 h following TBI. This study adheres to existing recommendations on diagnostic model development, individual patient data meta-analysis (IPD-MA), and reports the resulting model in accordance with the Transparent Reporting of a multivariable model for Individual Prognosis or Diagnosis (TRI-POD) guidelines (see TRIPOD checklist in Additional file 1: S3)[14]. Prospective data collection started in November 2011 and ended in December 2020.

### Participants

All patients, 18 years old or more and suspected of traumatic brain injury with a Glasgow coma score ≤ 12 and directly transported to a participating SNC. Patients who died within 24 h after admission or not directly admitted to a participant SNC were excluded. All trauma centers from the Traumabase® registry were included in this study (Additional file 1: S1).

### Data source

The Traumabase® registry prospectively collects socio-demographic, clinical, biological, therapeutic, and in hospital evolution data for all patients consecutively admitted to a participating center for a suspicion of severe trauma based on national triage criteria [15]. For each patient, data collection ranges from the prehospital scene to hospital discharge. Severe trauma is defined as a situation suggesting life threatening or changing injuries (Additional file 1: S2).

### Outcome measure

The primary outcome measure was the need for emergency neurosurgery (EN) within the 24 h following the traumacenter admission. Emergency neurosurgery was defined as subdural or epidural hematoma evacuation, intracerebral evacuation, decompressive craniectomy and external ventricular drainage for intracranial hypertension. The choice to perform EN was left to the trauma team in charge.

### Predictor selection

This work considered initially using all 39 prehospital variables available in the data base. Preliminary analysis using machine learning methodology showed that these variables to be contributive predictors but only a limited number of them were identified to be important to ensure a satisfactory model performance. Several iterations were conducted to select and retain the most relevant predictors according to the Shapley values. This process provided fifteen prehospital predictors:

(1) Glasgow coma score, (2) Initial systolic blood pressure, (3) Initial diastolic blood pressure, (4) Initial oxygen saturation, (5) Orotracheal intubation (during prehospital management), (6) Pupillary abnormalities (one or both pupils unreactive), (7) Administration of osmotherapy (Mannitol or Hypertonic Saline Solution (SSH) during prehospital management), (8) Regression of pupillary abnormality after osmotherapy, (9) Initial heart rate, (10) Mechanism of injury: fall from height, (11) Mechanism of injury: road traffic accident, (12) Mechanism of injury: firearm injury, (13) Mechanism of injury: other mechanisms, (14) Mechanism of injury: blunt by object (15) Capillary hemoglobin value.

### Data analysis

**Fig. 1:**
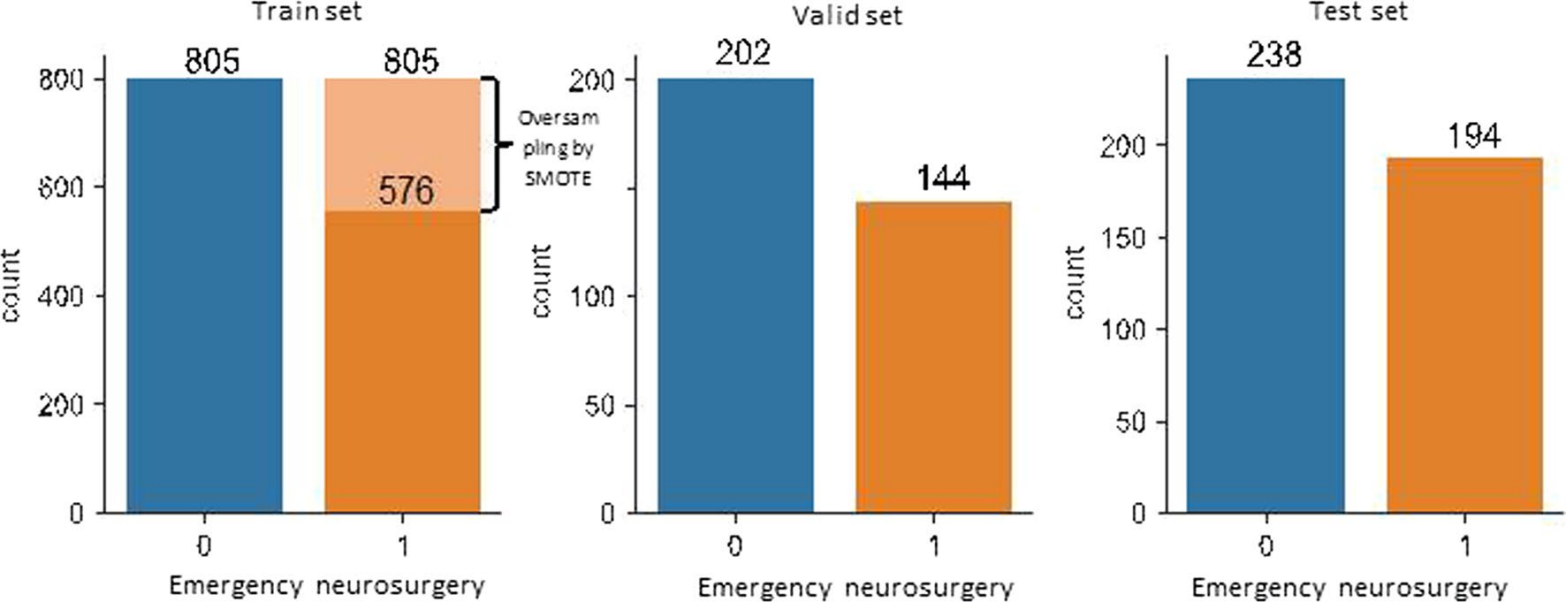
Distribution of train-valid-test dataset: “Synthetic” examples are created by Synthetic Minority Oversampling Technique (SMOTE) only in the training set to cope with the bias toward the majority class due to the imbalanced distribution of the target variable (Emergency neurosurgery within the 24 h hours after admission)

The dataset was divided into three partitions: the derivation set (training set and validation set) and the test set. Derivation and test set were composed according to a temporal split (derivation set from 15/11/2010 to 12/5/2019, the test set from 13/5/2019 to 6/12/2020). The derivation set was split again into training and validation sets based on the 80/20 time-based split. Missing values were handled by the factorial analysis for mixed data (FAMD) imputation strategy. Models trained with an imbalanced dataset risk a bias toward the majority class. To avoid this bias, the study group adopted the Synthetic Minority Over-sampling Technique (SMOTE) in order to balance class distribution in the training set [16] (Fig. 1). SMOTE randomly increases the minority class (need of neurosurgery after TBI) examples by synthesizing new minority instances from the existing ones. They are generated by randomly selecting one or more of the k-nearest neighbors for each example in the minority class.

Training proceeded on the training set and parameters defining the model architecture (hyperparameters) were determined with the validation set. Once the set of hyperparameters leading to the best model performance were determined, a final evaluation was carried out on the test set. This final step aimed to test out how the trained model could be generalized to the new data which correspond to the test set.

### Model selection

The performance of the prediction was compared among candidate models, which included both linear and nonlinear prediction models. These included logistic regression, k-nearest neighbors, stochastic gradient descent, and ensemble methods such as that of gradient boosting. In order to maximize the timely identification of patients that require EN, models were trained by optimizing an F2-score. This choice allowed the model to be trained by limiting false negatives responsible for potentially harmful secondary transfer delaying necessary EN. The mean Area Under Curve (AUC) was calculated to measure and compare the predictive performance of each model during the final step (test set).

### Interpretability

SHAP (Shapley Additive exPlanations) is a framework for Shapley Value which attributes an importance value to each for the prediction (emergency neurosurgery within 24 h in this work). The Shapley value provides a quantitative measure into how important each prehospital variable is to the overall cooperation of variable. For every patient, the Shapley value of each variable is calculated to identify its impact on the prediction of EN within 24 h. By averaging the Shapley value for each variable of each patient we were able to rank and evaluate its importance in the prediction model. The Shapley value works for both classification of variable importance and variable effects. A summary plot combines variable importance and effects. Each point on the summary plot is a Shapley value for a variable and an instance. The color represents the value of the variable from low (blue) to high (red), overlapping points are deterred in the y-axis. In this work, positive Shapley values contribute to the prediction of positive outcome (EN within 24 h) and vice versa.

### Statistical analysis

To describe the continuous variables, we applied median and interquartile range while number and proportion were applied for the categorical variables.

## Results

### Population

**Fig. 2:**
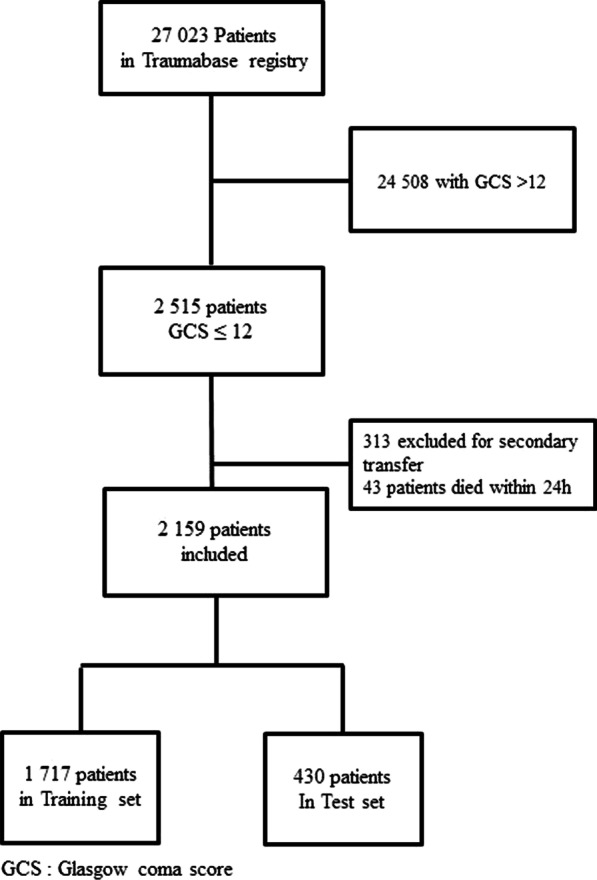
Flowchart

A total of 27,023 patients were included in the Traumabase® between November 2011 and December 2020. 2,159 patients meeting the inclusion criteria were included in the study of whom 914 patients (42%) required EN within the first 24 h (Flow chart, Fig. 2). The distribution of the number of patients included in the derivation set (training and validation set) and in the test set is presented in Fig. 2. The mean age was 35 years [IQR 24–52] and the population was predominantly male (77%). Road traffic accidents were the main cause of trauma (53%) followed by falls from height (32%). The median GCS score was 6 [3–9] and 36% had a pupillary abnormality. Injury severity score (ISS) was 29 [22–38] and 84% of the patients were on mechanical ventilation in the pre-hospital setting. The characteristics of the patients without imputation and after imputation are summarized in Table 1.

**Table 1:** Patient’s characteristics before and after imputation

	Unimputed		Imputed	
	Value	*n*	Value	*n*
Age, years	35 [24–52]	2159	35 [24–52]	2159
Female	494 (22.9)	2153	499 (23.1)	2159
BMI kg/m^2^	24 [22–26]	1829	24 [22–26]	2159
GCS	6 [3–9]	2159	6 [3–9]	2159
Initial oxygen saturation, %	97 [91–99]	1924	96 [90–99]	2159
Heart rate, beats per minute	91 [72–115]	1706	90 [75–110]	2159
Capillary hemoglobin value (HemoCue)	13.4 [12.0–14.9]	1717	13.0 [12.0–14.0]	2159
Initial blood pressure				
Systolic blood pressure	122 [100–140]	1709	121 [100–140)	2159
Diastolic blood pressure	75 [60–89]	1701	72 (60–86]	2159
Orotracheal intubation in prehospital setting, *n* (%)	1818 (84.7)	2147	1820 (84.3)	2159
Presence of pupillary abnormalities, *n* (%)	762 (35.7)	2137	770 (35.7)	2159
Administration of osmotherapy (Mannitol or HSS), *n* (%)	515 (23.9)	2159	515 (23.9)	2159
Regression of pupil abnormality after administration of osmotherapy, *n* (%)	228 (34.7)	657	228 (34.7)	657
Type of accident		2158		2159
Road traffic accident (%)	1150 (53.3)		1150 (53.3)	
Fall from height (%)	681 (31.6)		681 (31.6)	
Firearm (%)	118 (5.5)		118 (5.5)	
Hit by blunt object (%)	67 (3.1)		67 (3.1)	
Other	142 (6.6)		143 (6.6)	
ISS head-neck	4 [2–5]	2159	4 [2–5)	2159
ISS	29 [22–38]	2110	29 (22–38]	2159
SAPS 2	50 [39–63]	2132	50 (39–63]	2159
SOFA	9 [7–12)	1367	8 [7–11]	2159
In hospital mortality, *n* (%)	573 (27.7)	2159	573 (27.7)	2159

*BMI* body mass index, *GCS* Glasgow coma score, *ISS* injury severity score, *SAPS 2* simplified acute physiology score

### Prediction model

**Fig. 3:**
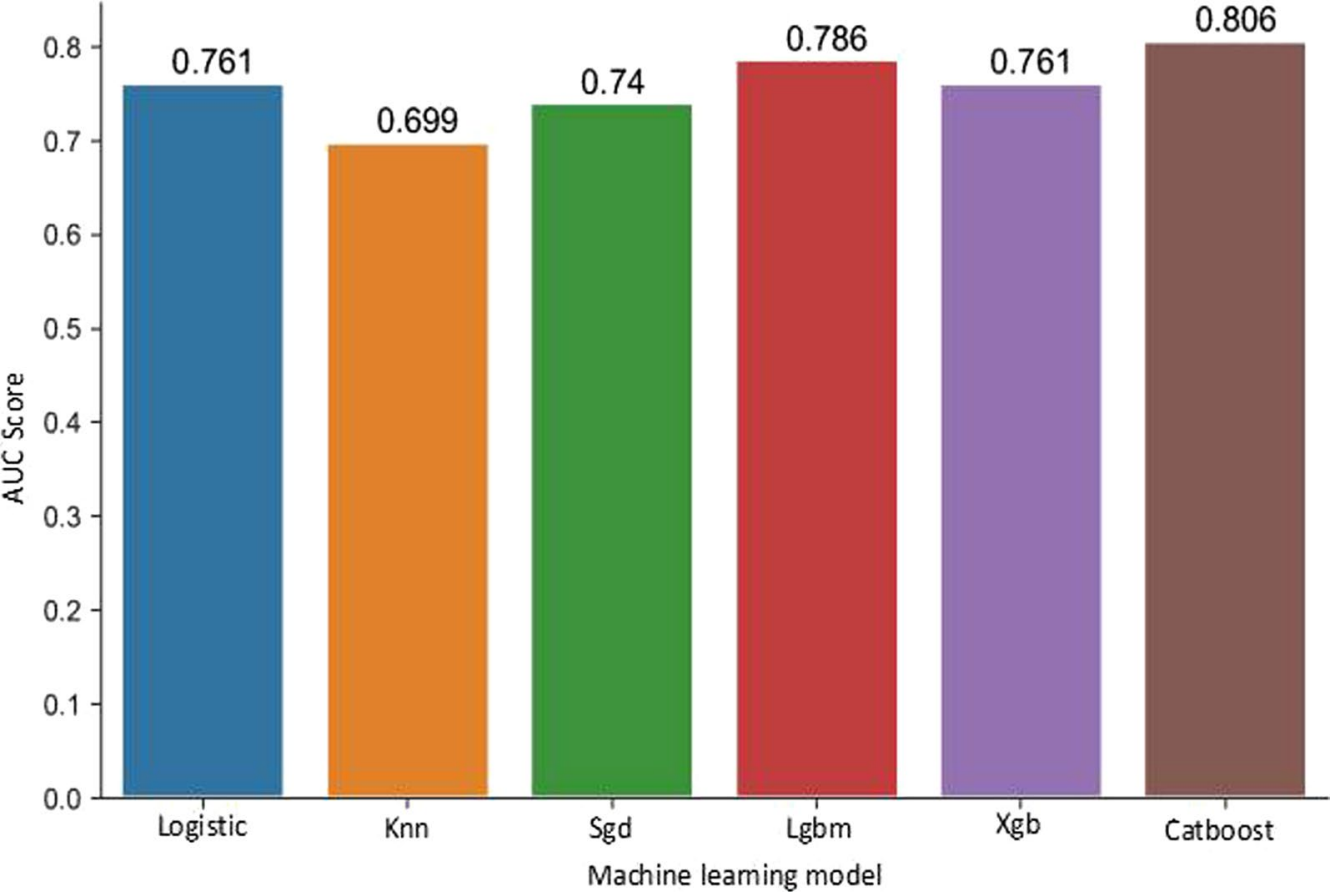
Area under the curve of the different artificial intelligence models after the "Test set" phase

The logistic regression model showed an AUC of 0.76, the nearest neighbor (knn) technique performed less favorably with an AUC of 0.70. The AUCs of the other set of techniques (Logistic regression, Knn, Sgd, Lgbm and xgb) ranged from 0.70 to 0.81 (Fig. 3). The best predictive model for EN within the first 24 h was obtained with the CatBoost set technique (AUC: 0.81) (Fig. 3).

### Confusion matrices

**Fig. 4:**
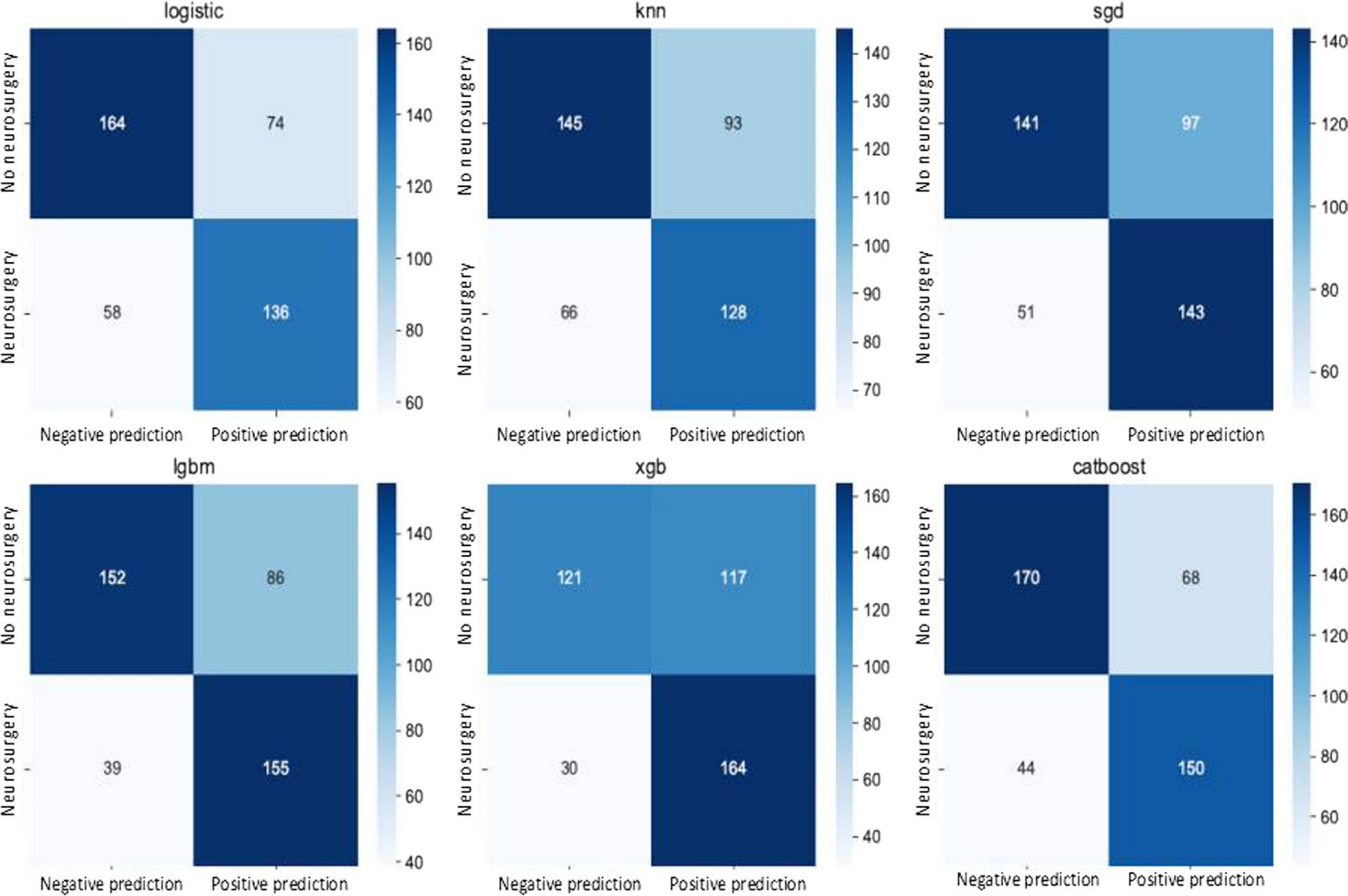
Matrice of confusion of the models: The confusion matrix describes the performance of each classification model. For example, the Catboost model has a balanced prediction with 44 false negatives (patient that will require neurosurgery but not identified by the model) and 68 false positives (patient that will not require neurosurgery but identified by the model as requiring emergency neurosurgery)

The confusion matrices for the different models are presented in Fig. 4. All the models trend to limit the number of false negatives. The Catboost model has a balanced prediction with 44 false negatives and 68 false positives. The best predictive model to limit the false negative is the xgb (30 false negatives) (Fig. 4).

### Predictor selection

**Fig. 5:**
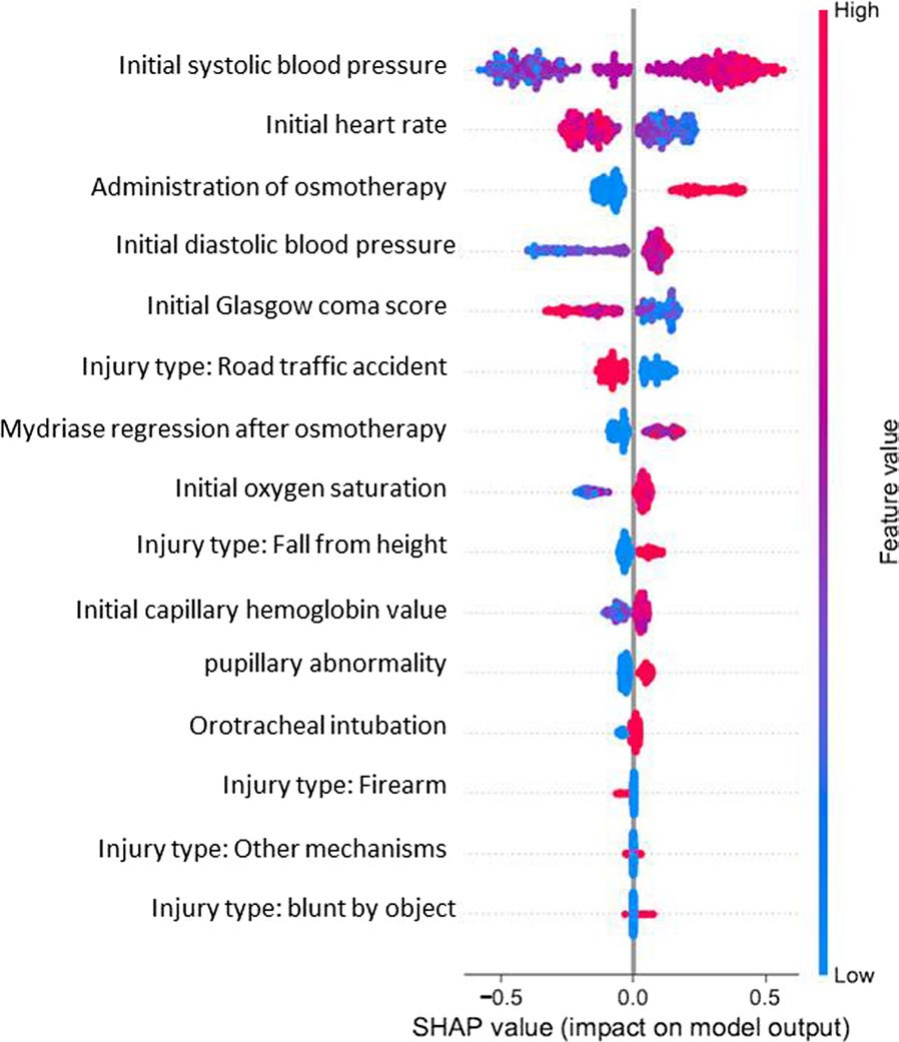
Shapley values of the Catboost model: each point on the summary plot is a Shapley value for a variable and an instance. The color represents the value of the variable from low (blue) to high (red). The most important variable that helps in the prediction is the systolic pressure upon arrival of the physician-staffed EMS and the less important is. For example, the distribution of the feature value along the x-axis indicates that low systolic pressure contributes to a prediction of negative outcome and high systolic pressure contributes to a prediction of positive outcome

According to the Shapley values, of the 15 prehospital variables used by the model, a low Glasgow Coma Score, the regression of the mydriasis after osmotherapy, a high systolic or diastolic blood pressure and a low heart rate were the most influential variables to predict neurosurgery within 24 h following trauma (Fig. 5). All the prehospital variables used in the model are presented with the Shapley value in Fig. 5.

## Discussion

Prehospital identification of TBI patients who require EN within 24 h could be useful to increase timely disposition to a SNC, to efficiently anticipate required resources and accelerate a patient specific workflow. In the present study, ML-based models were able to predict with sufficient discrimination the necessity for EN within 24 h after moderate and severe TBI. This result tends to confirm that linear models such as CatBoost convey new tools to elaborate prediction model as compared to commonly used logistic regression models (Additional file 2: Graphical abstract).

This study has several strengths. Firstly, data were extracted from a large national registry with robust data management. These data were collected from multiple geographically and structurally distinct regions with different trauma systems. Secondly, data were analyzed by trained and experienced data scientists through a multi-disciplinary partnership [17]. Thirdly, whereas machine learning-based models frequently rely on a large number of variables the present models rely exclusively on a few, rapidly and easily available pre-hospital variables in daily practice and make it suitable for a decision-making tool. Fourthly, our results are consistent with a recent study by Abe et al. In this study, ML algorithms have achieved the same performance as our model (AUC: 0.78) in detecting intracerebral hemorrhage after traumatic brain injury [18]. Finally, this study provides information on the “black box” of the model through Shapley values in order to ensure explainability for clinicians and increase acceptance.

### Predictor selection

ML-based decision support systems frequently lack the possibility to be understood in their logic. Explainability and interpretability of ML models remain a challenge in the field of data science and should be improved in order to provide fully accurate and convincing results. In our study, the “black box” of the most pertinent model (Catboost model) was explored through the Shapley value. This method provides an explanation to the mechanism of a complex model and illustrates how decisions are made. This insight confirms to the clinicians that ML apply to medical data present a medical consistency. In the Catboost model, the most important variable in the prediction was the SBP with more EN use when SBP is high. Moreover, a low heart rate value was associated with neurosurgery. The association of a high blood pressure and a low heart rate known as the Cushing's reflex corresponds to the adaptive response of the brain to severe intracranial hypertension [19, 20]. It has already been demonstrated that this reflex was a predictor for neurosurgery [21]. A low Glasgow Coma Score and a pupillary abnormality were found to be important factors for EN prediction. They frequently indicate a severe brain lesion such as subdural or extradural hematoma with a mass effect on the brain [22]. All the variables, but also the meaning of the variables in our predictive model seem to be medically consistent and present a strong physiopathological rationale.

### Outcome: emergency neurosurgery within 24 h after admission

ML techniques, especially supervised learning techniques, optimize their models based on large data sets. Thus, the definition of the prediction target is of crucial importance. In the registry used for this study, EN within 24 h is an observed and predefined variable and is not derived from a theoretical indication or proxy. This specificity may increase inaccuracy if the model is applied to datasets that rely on theoretical indication or a proxy. Furthermore, clinical practice patterns may vary across clinicians, hospitals or trauma systems [23]. For example, based to the Shapley values, TBI after firearm injury have less neurosurgical requirement than other mechanisms. We can hypothesize, in these cases, that clinicians agreed on early withdrawal of care and refrained from surgical management of often catastrophic ballistic cerebral injuries. Thus, the self-learning quality of the model may induce a systemic bias in this case. To further compensate for the bias of early withdrawal of care in the case of catastrophic injury, all patients who died within 24 h were excluded. The 42% rate of EN in our study is higher than the 15–30% reported in previous studies. Those studies generally focus on the admission period, whereas the present study extended to the first 24 h during which injuries such as contusions or subdural and extradural hematomas can evolve and required secondary neurosurgical management.

### Perspective

Referral of trauma patients to a trauma center improves mortality and morbidity, especially when the severity of the injury is significant [24, 25]. Indeed, trauma systems can only reach their full potential when patients are transported to the right hospitals within the right time and if local resources are activated appropriately. Thus, early identification of TBI patients that will require EN is important to avoid delay or anticipate adequate treatment (blood product for TBI coagulopathy, catecholamine to improve cerebral pressure perfusion). However, the prehospital triage phase is time-constrained and aids to diagnosis are limited. Hence, this ML model which is based exclusively on a few pre-hospital variables could help to streamline the entire management of the most severe TBI patients with the final purpose to improve early survival and reduce long-term disabilities [8, 26, 27].

ML-based clinical algorithms have to be focused on augmenting rather than replacing human intelligence. In situations characterized by uncertainty and complexity, ML tools could provide more reliable and reproducible decision-making or support human decision-making when in doubt. This hypothesis needs to be tested. The fact that our model used the same physiological reasoning as clinicians confirms its robustness but also raises the question of its added value. In consequence, the next step is to assess prospectively the performance of the tool compared to physician prediction based on the same information. Finally, if ML models are used in a daily practice to detect the severity of patients’ injuries and for decision-making process, their safety profiles should be carefully evaluated in order to avoid harming a group of patients. Indeed, as presented in confusion matrix, the Catboost model had a 20% false negative rate for NS. This false negative rate could be a concern at the patient level considering the risk of undertriaged and therefore denied access to high-resource resuscitation. Thus, the final step requires work flow integration and assessment of interaction with the clinicians and patient’s outcome.

### Limitation

The limitations of this study require consideration. Firstly, this is a retrospective study based on registry data and we were not able to breakdown the type of brain injuries (subdural hematoma, epidural hematoma, subarachnoid hemorrhage). Secondly, the study focused exclusively on directly admitted patients to exclude cases with missing data, since reliable prehospital data are often missing after secondary transport. Thirdly, the SMOTE technique employed to balance each group during the training phase may generate bias by creating artificial new patients. However, the technique remains a recommended reference frequently used in ML models. Fourthly, Shapley values do not provide any specific thresholds for continuous variables such as high blood pressure or low heart rate. Thus, we are unable to recommend a precise threshold for each feature to trigger the referral of TBI patients to a specialized center. Finally, to design this model we have chosen a time-based split which is questionable considering the evolution in TBI management and prognosis in recent years. However, to frame time-dependent events and dynamic change such as management patterns, time-based splitting provides a robust and recommended approach.

## Conclusion

In this study several ML models predicted the necessity for EN within 24 h after moderate and severe TBI with acceptable discrimination. These models are based exclusively on a few, rapidly and easily available pre-hospital variables opening the perspective to integrate the model easily into a clinical decision support tool. The reliability and usefulness of such a tool deserves assessment in prospective studies.

## Supplementary Information

**Additional file 1:** Supplementary materials.

**Additional file 2:** Graphical abstract.

## Data Availability

The data that support the findings of this study are available from the Traumabase group but restrictions apply to the availability of these data, which were used under license for the current study, and so are not publicly available. Data are however available from the authors upon reasonable request and with permission of the Traumabase® group.

## Abbreviations

TBI: Traumatic brain injury
SNC: Specialized neurotrauma center
EN: Emergency neurosurgery
SMOTE: Synthetic minority over-sampling technique
GCS: Glasgow coma score
AUC: Area under the curve
ISS: Injury severity score
