# Supplementary material for: Machine learning-based prediction of emergency neurosurgery within 24 h after moderate to severe traumatic brain injury
Source: World J Emerg Surg. 2022 Aug 3;17:42. doi: 10.1186/s13017-022-00449-5 (PMC9351267; doi:10.1186/s13017-022-00449-5)
Supplement: Supplementary file 1 — Additional file 1: Supplementary materials. [file 13017_2022_449_MOESM1_ESM.docx]

**Machine Learning Based Prediction of Emergency Neurosurgery within 24 hours after Moderate to Severe Traumatic Brain Injury**

Supplementary materials 1

1.Department of Anesthesiology and Critical Care, AP-HP Beaujon,

2. Department of Anesthesiology and Critical Care, AP-HP Bicétre

3.Department of Anesthesiology and Critical Care, AP-HP Hopital Européen Georges Pompidou

4.Department of Anesthesiology and Critical Care, AP-HP Mondor

5.Department of Anesthesiology and Critical Care, AP-HP Pitié-Salpêtrière

6. Department of Anesthesiology and Critical Care Metz Thionville

7.Department of Anesthesiology and Critical Care, CHU Nancy

8.Department of Anesthesiology and Critical Care, CHU Toulouse Purpan

9.Department of Anesthesiology and Critical Care, CHU Toulouse Rangeuil

10.Department of Anesthesiology and Critical Care, CHU Caen

11.Department of Anesthesiology and Critical Care, CHU Reims

12.Department of Anesthesiology and Critical Care, CHU Rouen

13.Department of Anesthesiology and Critical Care, HIA, Percy

14.Department of Anesthesiology and Critical Care, HIA Saint-Anne

15.Department of Anesthesiology and Critical Care, Hopital Civil de Colmar

16. Department of Anesthesiology and Critical Care, CHU Bordeaux

17. Department of Anesthesiology and Critical Care, CH de Valenciennes

**Supplementary materials 2:** *Vittel Criteria*

**Step 1 (Physiological signs)**

GCS < 13

SAP < 90 mmHg

SpO2 < 90%

**Step 2 (Global assessment of speed and mechanism)**

Ejection from vehicle

Death in same passenger compartment

Fail > 6 m

Victim thrown or projected

Global assessment of speed and potential injuries:

Vehicle deformation, estimated vehicle speed no helmet, no seat belt

Blast

**Step 3 (Anatomical injuries)**

Penetrating trauma of head, neck, thorax, abdomen, arms or legs

Flail chest

Severe burn

Pelvic fracture

Suspicion of spinal cord injury

Amputation at or above wrist or ankle level

Acute limb ischemia

**Step 4 (resuscitation)**

Mechanical ventilation

Intravascular filling > 1000 ml

Vasopressor

**Supplementary materials 3:** check list

| **Section/Topic** | **Item** | **Checklist Item** | **Page** |
| --- | --- | --- | --- |
| **Title and abstract** | | | |
| Title | 1 | Identify the study as developing and/or validating a multivariable prediction model, the target population, and the outcome to be predicted. | 1 |
| Abstract | 2 | Provide a summary of objectives, study design, setting, participants, sample size, predictors, outcome, statistical analysis, results, and conclusions. | 3-4 |
| **Introduction** | | | |
| Background and objectives | 3a | Explain the medical context (including whether diagnostic or prognostic) and rationale for developing or validating the multivariable prediction model, including references to existing models. | 5 |
|  | 3b | Specify the objectives, including whether the study describes the development or validation of the model or both. | 5 |
| **Methods** | | | |
| Source of data | 4a | Describe the study design or source of data (e.g., randomized trial, cohort, or registry data), separately for the development and validation data sets, if applicable. | 6 |
|  | 4b | Specify the key study dates, including start of accrual; end of accrual; and, if applicable, end of follow-up. | 6 |
| Participants | 5a | Specify key elements of the study setting (e.g., primary care, secondary care, general population) including number and location of centres. | 6 |
|  | 5b | Describe eligibility criteria for participants. | 6 |
|  | 5c | Give details of treatments received, if relevant. | - |
| Outcome | 6a | Clearly define the outcome that is predicted by the prediction model, including how and when assessed. | 6-7 |
|  | 6b | Report any actions to blind assessment of the outcome to be predicted. | - |
| Predictors | 7a | Clearly define all predictors used in developing or validating the multivariable prediction model, including how and when they were measured. | 7 |
|  | 7b | Report any actions to blind assessment of predictors for the outcome and other predictors. | - |
| Sample size | 8 | Explain how the study size was arrived at. | 10-11 |
| Missing data | 9 | Describe how missing data were handled (e.g., complete-case analysis, single imputation, multiple imputation) with details of any imputation method. | 7 |
| Statistical analysis methods | 10a | Describe how predictors were handled in the analyses. | 8 |
|  | 10b | Specify type of model, all model-building procedures (including any predictor selection), and method for internal validation. | 8-9 |
|  | 10d | Specify all measures used to assess model performance and, if relevant, to compare multiple models. | 8-9 |
| Risk groups | 11 | Provide details on how risk groups were created, if done. | - |
| **Results** | | | |
| Participants | 13a | Describe the flow of participants through the study, including the number of participants with and without the outcome and, if applicable, a summary of the follow-up time. A diagram may be helpful. | 11 |
|  | 13b | Describe the characteristics of the participants (basic demographics, clinical features, available predictors), including the number of participants with missing data for predictors and outcome. | 10 and12 |
| Model development | 14a | Specify the number of participants and outcome events in each analysis. | 10-12 |
|  | 14b | If done, report the unadjusted association between each candidate predictor and outcome. | - |
| Model specification | 15a | Present the full prediction model to allow predictions for individuals (i.e., all regression coefficients, and model intercept or baseline survival at a given time point). | - |
|  | 15b | Explain how to the use the prediction model. |  |
| Model performance | 16 | Report performance measures (with CIs) for the prediction model. | 13 |
| **Discussion** | | | |
| Limitations | 18 | Discuss any limitations of the study (such as nonrepresentative sample, few events per predictor, missing data). | 15-16 |
| Interpretation | 19b | Give an overall interpretation of the results, considering objectives, limitations, and results from similar studies, and other relevant evidence. | 13-14 |
| Implications | 20 | Discuss the potential clinical use of the model and implications for future research. | 15-16 |
| **Other information** | | | |
| Supplementary information | 21 | Provide information about the availability of supplementary resources, such as study protocol, Web calculator, and data sets. | 20 |
| Funding | 22 | Give the source of funding and the role of the funders for the present study. | 23 |
