# Supplementary material for: Machine learning-based prediction of emergency neurosurgery within 24 h after moderate to severe traumatic brain injury
Source: World J Emerg Surg. 2022 Aug 3;17:42. doi: 10.1186/s13017-022-00449-5 (PMC9351267; doi:10.1186/s13017-022-00449-5)
Supplement: Supplementary file 2 — Additional file 2: Graphical abstract. [file 13017_2022_449_MOESM2_ESM.pptx]

## Slide 1
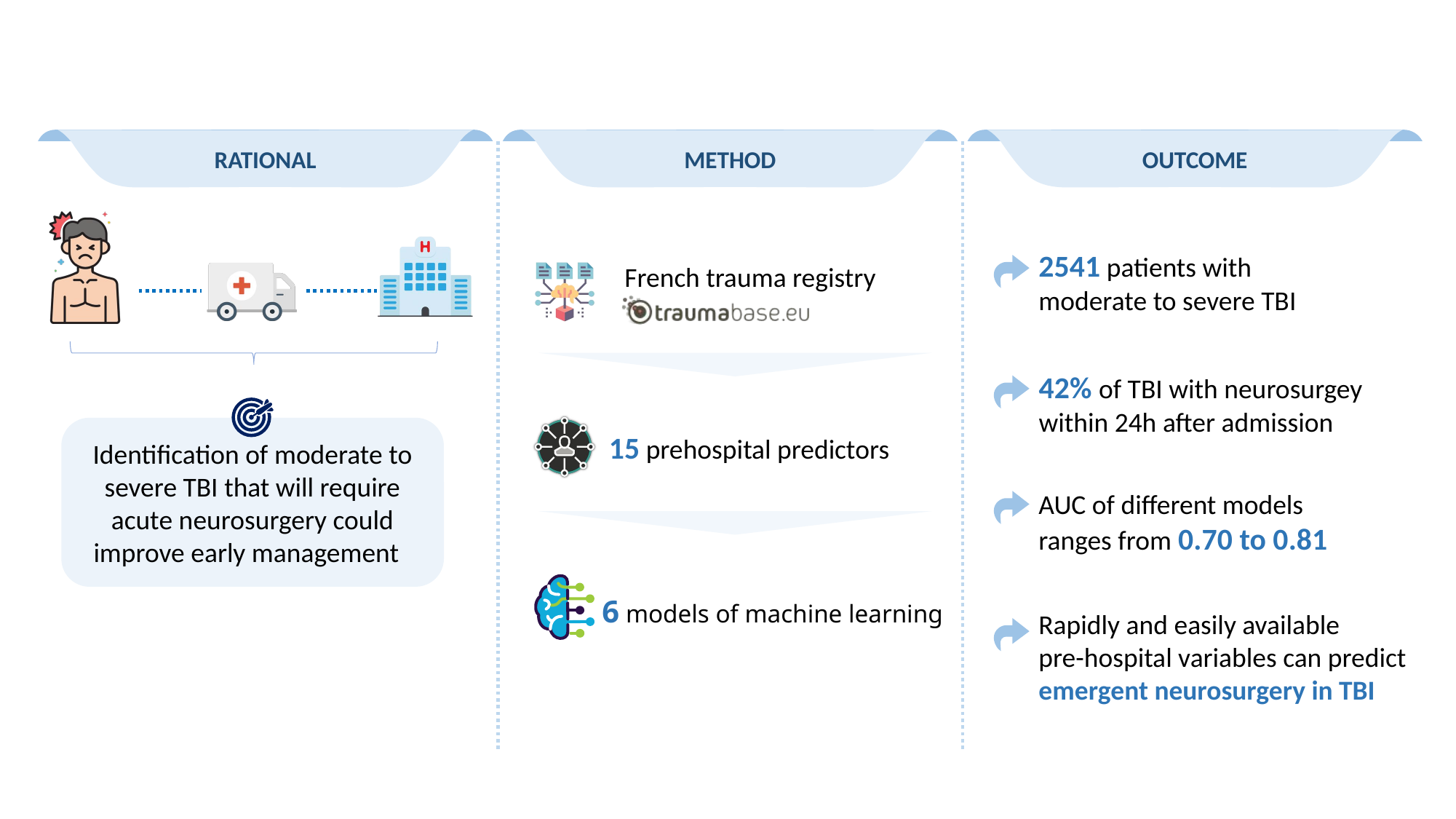

RATIONAL
METHOD
OUTCOME
2541 patients with
moderate to severe TBI
French trauma registry
42% of TBI with neurosurgey within 24h after admission
Identification of moderate to severe TBI that will require acute neurosurgery could improve early management
15 prehospital predictors
AUC of different models
ranges from 0.70 to 0.81
6 models of machine learning
Rapidly and easily available
pre-hospital variables can predict emergent neurosurgery in TBI
